# Dapagliflozin promotes browning of white adipose tissue through the FGFR1-LKB1-AMPK signaling pathway

**DOI:** 10.1007/s11033-024-09540-3

**Published:** 2024-04-21

**Authors:** Yue Lv, Chengrui Zhao, Qiuyan Jiang, Yilin Rong, Mingfeng Ma, Lili Liang, Weiping Li, Jiuxuan Zhang, Ning Xu, Huiwen Wu

**Affiliations:** 1 Science and Technology Center of Fenyang College, Shanxi Medical University, No. 16 Xueyuan Road, Fenyang, Shanxi 032200 People’s Republic of China; 2 Cultivation Key Laboratory of Metabolic Cardiovascular Diseases Research, Fenyang, 032200 People’s Republic of China; 3 Basic Sciences Department of Fenyang College, Shanxi Medical University, Fenyang, 032200 People’s Republic of China; 4 Department of Oncology, Shanxi Province Fenyang Hospital, Fenyang, 032200 People’s Republic of China

**Keywords:** Dapagliflozin, Obesity, White adipose tissue browning, FGFR1, LKB1, AMPK

## Abstract

**Background:**

Obesity is associated with a wide variety of metabolic disorders that impose significant burdens on patients and society. The “browning” phenomenon in white adipose tissue (WAT) has emerged as a promising therapeutic strategy to combat metabolic disturbances. However, though the anti-diabetic drug dapagliflozin (DAPA) is thought to promote “browning,” the specific mechanism of this was previously unclear.

**Methods:**

In this study, C57BL/6 J male mice were used to establish an obesity model by high-fat diet feeding, and 3T3-L1 cells were used to induce mature adipocytes and to explore the role and mechanism of DAPA in “browning” through a combination of in vitro and in vivo experiments.

**Results:**

The results show that DAPA promotes WAT "browning" and improves metabolic disorders. Furthermore, we discovered that DAPA activated "browning" through the fibroblast growth factor receptors 1-liver kinase B1-adenosine monophosphate-activated protein kinase signaling pathway.

**Conclusion:**

These findings provide a rational basis for the use of DAPA in treating obesity by promoting the browning of white adipose tissue.

## Introduction

Obesity poses a pressing global health concern and leads to severe consequences such as metabolic diseases [1]. The underlying cause of obesity is an imbalance between energy intake and expenditure in adipose tissue. As mammals, human beings have two types of adipose tissue: white adipose tissue (WAT), which is the main energy storage organ in the body; and brown adipose tissue (BAT) [2], which, due to its high content of uncoupling protein-1 (UCP1), can uncouple ATP to facilitate energy consumption. However, recent research has revealed an intriguing finding: WAT undergoes a transformation process known as browning, converting it into beige adipose tissue (or “Brite”). This newly identified type of adipose tissue also plays a critical role in regulating energy metabolism. Moreover, increasing evidence suggests that the browning of WAT and activation of BAT can ameliorate metabolic disorders, bringing wide attention to these phenomena as methods to combat obesity [3–5].

Fibroblast growth factor 21 (FGF21) is a nonclassical fibroblast growth factor that also plays a role in the treatment of metabolic disorders. It is primarily secreted by the liver in response to a ketogenic diet and fasting, and FGF21 signaling requires an interaction between fibroblast growth factor receptors (FGFR) and the β-Klotho (KLB) complex [6]. However, in the research of Leiluo Geng et al. [7], it was found that obesity can lead to an increase in FGF21 levels, but it did not show its protective effect on metabolic disorders. They called this “FGF21 resistance,” and their research also proved that “FGF21 resistance” may be related to the decrease of FGFR and KLB. Adipose tissue expresses both FGFR and KLB [8], with FGFR1 being the main one. Studies have shown that long-term high-fat diet feeding significantly reduces FGFR1 and KLB in mice [9]. Christopher J Lelliott et al. [10] have shown that targeting FGFR1 can improve obesity and glucose intolerance by activating the FGFR1 pathway. Thus, it can be seen from this that activating the FGFR1/KLB-mediated signaling cascade is very important for improving metabolic disorders, but the specific mechanism is still unknown.

Emerging evidence also suggests that dapagliflozin (DAPA), a widely prescribed medication for diabetes, has potential protective effects on the heart and kidneys and alleviates metabolic disorders in various ways [11–13]. The latter effect was observed in studies investigating the treatment of metabolic syndrome, which established obesity models in rodents and treated them with DAPA. The therapeutic effects of DAPA on metabolism appear to be associated with WAT browning [14]. Specifically, following DAPA treatment, obese mice fed a high-fat diet experienced weight loss, primarily through reduction in subcutaneous and visceral fat pools. Additionally, DAPA treatment promotes mitochondrial biogenesis and increases cellular energy expenditure [15, 16]. Notably, the expression of UCP1 in both WAT and BAT was significantly elevated, indicating DAPA’s ability to promote the activation of BAT and the “browning” of WAT [14]. Collectively, these effects contribute to the regulation of metabolic disorders. We thus hypothesized that DAPA could induce WAT browning, though the precise mechanisms underlying DAPA’s promotion of browning remain unclear.

Adenosine monophosphate-activated protein kinase (AMPK), a crucial regulator of lipid metabolism and an energy sensor, also plays a significant role in the “browning” process [17]. AMPK responds to sympathetic signaling, various kinases, and compounds to promote BAT biogenesis, increase energy expenditure, and induce the browning of white adipocytes in vivo [18]. Previous studies investigating the relationship between BAT and AMPK have highlighted the impact of various AMPK signaling pathways on BAT, with the liver kinase B1 (LKB1)/AMPK pathway being particularly important [19]. Research conducted by Jing Yi Luo et al. [20] showed that DAPA primarily improves hepatic steatosis by activating AMPK and increasing LKB1 expression in the liver. However, the specific role of DAPA in browning and the associated signaling pathways remain unclear. Further investigation is thus needed to clarify the mechanisms through which DAPA promotes browning.

This study aimed to replicate an obese mouse model (C57BL/6 J) using a high-fat diet (HFD). By conducting in vivo and in vitro experiments, we investigated the role and signaling pathways in the browning process promoted by shedding light on the mechanism by which DAPA promotes the browning of adipose tissue in obese mice.

## Materials and methods

### Materials

The replication of the obese model necessitated the obtainment of materials from various sources. Fetal bovine serum (FBS; PYG0001), Dulbecco’s modified Eagle’s medium (DMEM; PYG0073), dimethyl sulfoxide (DMSO; PYG0040), an ELC western blot detection kit (AR1171), penicillin/streptomycin (PYG0016), and trypsin (PYG0015) were all purchased from Boster Biotechnology (USA). An Oil Red O staining kit (#1262) and Mito Tracker Red CMXRos (M9940) were purchased from Solarbio (China). A RIPA lysis buffer (HY-K1001), insulin (HY-P1156), dexamethasone (DEX; HY-14648), dapagliflozin (HY-10450), PD173074 (HY-10321), Compound C (HY-13418A) and Pim1/AKK1-IN-1 (HY-10371) were purchased from MedChemExpress (USA). Isobutylmethylxanthine (IBMX; I7018) was purchased from Sigma-Aldrich (USA).

Various antibodies were also obtained. p-FGFR1 (AF8210), p-LKB1 (AF8186), and p-AMPK (AF3423) were purchased from Affinity (China). AMPK (BM4202) and LKB1 (PB0257) were purchased from Boster Biotechnology. β-actin (AC038), UCP1 (A5857), FGFR1 (A21219), and KLB (A12028) were purchased from ABclonal (China). Mouse Tumor Necrosis Factor-α(TNF-α) ELISA Kit (MU30030) and Mouse Interleukin6(IL-6) ELISA Kit (MU30044) were purchased from Bioswamp (China).

### Experimental animals

Four-week-old male C57BL/6 mice were purchased from the Shanxi People’s Hospital (Taiyuan, China). These experimental animals were reared in cages with a temperature of 22–24℃, 50% humidity, and a light–dark cycle of 12 h, with free access to food and water. Provided sufficient food to the mice every morning at 8 o’clock and recorded the weight of the food. Weighed the remaining food at the same time the next day and repeated this process until the end of treatment. Calculated the calories consumed based on the amount of food intake. The mice were randomly divided into three groups (n = 6): (1) normal diet (ND) + injection of physiological saline, (2) HFD containing 60% kilocaloric fat (Medicine Ltd., Yangzhou, China) + injection of physiological saline, and (3) HFD containing 60% kilocaloric fat + injection of DAPA (HFD + DAPA, 1 mg/kg/day) [21, 22]. Treatment was initiated in the 5th week and ended in the 10th week. Mice were weighed weekly, blood was collected from their orbital cavities, and they were immediately euthanized by intraperitoneal injection of pentobarbital after the experiment. Liver, epididymal white adipose tissue (eWAT), inguinal white adipose tissue (iWAT), and BAT were collected from each mouse for mRNA and western blot experiments.

### Cell culture

3T3-L1 cells were purchased from the National Cell Resource Sharing Platform (1101MOU-PUMC000155, Beijing, China) and cultured in a basic growth medium (DMEM containing 10 fetal bovine serum and 1% penicillin/streptomycin) at 37 ℃ and 5% CO2. To initiate differentiation, fully confluent cells (defined as day 0) were treated with induction medium I (containing 10 μg/mL insulin, 0.5 mM IBMX, and 1 μM DEX) for 2 days. After 48 h, induction medium II (containing 10 μg/mL insulin) was used for an additional 2 days. Thereafter, the basic growth medium was replaced every other day for a total of 4 days (until the 8th day). Process mature adipocytes with DAPA for 48 h; when using FGFR1, LKB1, and AMPK inhibitors, treat cells with inhibitors for 2 h first, then add DAPA for a total of 48 h of treatment. DMSO was used as the vehicle for all treatments.

### Histology and immunohistochemistry

The liver and adipose tissue collected immediately after the euthanasia of the mice was fixed in 4% paraformaldehyde, dehydrated, embedded in paraffin, and cut into 5-μm thick sections for histological analysis using hematoxylin and eosin (H&E) staining. Immunohistochemistry was used to detect the UCP1 content in the adipose tissue.

### Plasma biochemical measurements

Total cholesterol (TC, A111-1–1), total triglycerides (TG, A110-1–1), alanine aminotransferase (ALT, C009-2–1), and aspartate aminotransferase (AST, C010-2–1) levels in plasma were measured using commercial reagent kits from the Nanjing Construction Institute of Bioengineering Research (Nanjing, China).

### Mitochondrial labelling

The cells were treated with different methods, and then the basal culture medium was heated to 37 °C. Next, the MitoTracker probe solution (M9940, Solarbio) was added to a final concentration of 100 nM, following the manufacturer’s recommendations. The cells were then incubated with the staining solution for 30 min. Next, the cell nuclei were stained with DAPI (AR1176, Boster Biotechnology). Cells were observed and photographed using a fluorescent microscope.

### Immunofluorescence

Seed cells at a density of 10^5^ cells per plate were treated using different methods. The treated cells were fixed using 4% paraformaldehyde in the cold for 20 min, followed by permeabilization using 0.2% TritonX-100 at 4℃ for 10 min. They were then blocked with PBS containing 5% BSA, and incubated with UCP1 primary antibody at a 1:200 dilution overnight at 4℃. Finally, the treated cells were incubated with fluorescent secondary antibodies (1:50, BA1127, Boster Biotechnology) at room temperature for 2 h. After staining with DAPI for 3 min, the fluorescence intensity was observed using a fluorescence microscope, and photographs were taken.

### Cell cytotoxicity assay

3T3-L1 cells were seeded in a 96-well plate (701,001, NEST) at a density of approximately 1500 cells per well. The cells were treated with different DAPA concentrations After 48 h, 10 μL CCK-8 (AR1199, Boster Biotechnology) solution was added into each well and incubated at 37℃ for 2 h. The absorbance (A) balance was measured at 450 nm by the plate reader (AMG-2201–025, BioTek).

### Oil red O staining

3T3-L1 cells were seeded onto a plate, and Oil Red O staining was used to detect intracellular lipid accumulation according to the manufacturer’s instructions.

### Western blotting

Cells and tissues were collected and lysated using RIPA buffer (100:1:1 ratio of phosphatase inhibitor to protease inhibitor). After 30 min of lysis on ice, the total protein was extracted. After separation by SDS-PAGE (AR0138, Boster Biotechnology), the protein was transferred onto a nitrocellulose membrane (AR0135-04, Boster Biotechnology). The cells and tissues were then blocked with 5% skim milk at room temperature for 2 h and incubated with an appropriate concentration of primary antibody overnight at 4 °C. Finally, the membranes were incubated with peroxidase-conjugated goat anti-rabbit IgG (H + L) antibody (abs20002ss; Absin Bioscience) at room temperature for 2 h. The immunoreactive bands were detected using western blotting.

### GTT

After a 16-h fasting period, the fasting blood glucose levels (defined as 0 min) of the mice were measured. Then, a 20% glucose solution (1 g/kg) was injected into the abdominal cavity, and blood glucose levels were measured at 30, 60, 90, and 120 min. Blood samples were collected from the tail vein for all measurements. Data were recorded and a line graph was plotted.

### Real-time quantitative PCR

Total RNA was extracted from the collected tissue or cells using the TRIzol reagent (DP424, Tiangen). Next, cDNA was synthesized using the M5 Sprint qPCR RT kit with gDNA remover (MF949, Polymerase Limited Company, Beijing) according to the manufacturer’s instructions. qPCR was then performed using 2 × M5 HiPer SYBR Premix EsTaq (with Tli RNase H) (MF797-01, Polymerase Limited Company, Beijing). Finally, the 2^−ΔΔCT^ method was used to determine the expression of the target gene with β-actin as the internal reference. The primers used for the target genes are listed in Table 1, All primers were designed and synthesized by Sangon Biotech (Shanghai, China).

**Table 1:** The primer sequences of the target genes

Gene	Primer sequence 5' to 3'
*Cox7a1*	Forward, CCGTGTGGCAGAGAAGCAGAAG Reverse, GCCCAGCCCAAGCAGTATAAGC
*Cox8b*	Forward, CCCCTATCCTGCGGCTGCTC Reverse, CGGCGGAAGTGGGAGTTTTGG
*PPARγ*	Forward, TGTTCGCCAAGGTGCTCCAG Reverse, TGAAGGCTCATGTCTGTCTCTGTC
*Ucp-1*	Forward, AAACACCTGCCTCTCTCGGAAAC Reverse, TGCATTCTGACCTTCACGACCTC
*Pgc-1α*	Forward, GCATGACCCTCCTCACACCAAAC Reverse, TTGCGACTGCGGTTGTGTATGG
*C/EBPα*	Forward, GCTGAGCGACGAGTACAAGATGC Reverse, CTTGTGCTGCGTCTCCAGGTTG
*β-actin*	Forward, GTGCTATGTTGCTCTAGACTTCG Reverse, ATGCCACAGGATTCCATACC

### ELISA

Prepare adipose tissue into tissue homogenate and detect the content of inflammatory factors according to the instructions of the reagent kit.

### Statistical analysis

Data were expressed as mean ± SD and independent samples were statistically analyzed using the Student’s t-test or one-way ANOVA. In all analyses, *p* < 0.05 indicates a statistically significant difference. GraphPad Prism 8.3.0 (GraphPad Software, USA) is used for all analyses.

## Results

### DAPA shown to improve obesity induced in HFD-fed mice

**Fig. 1:**
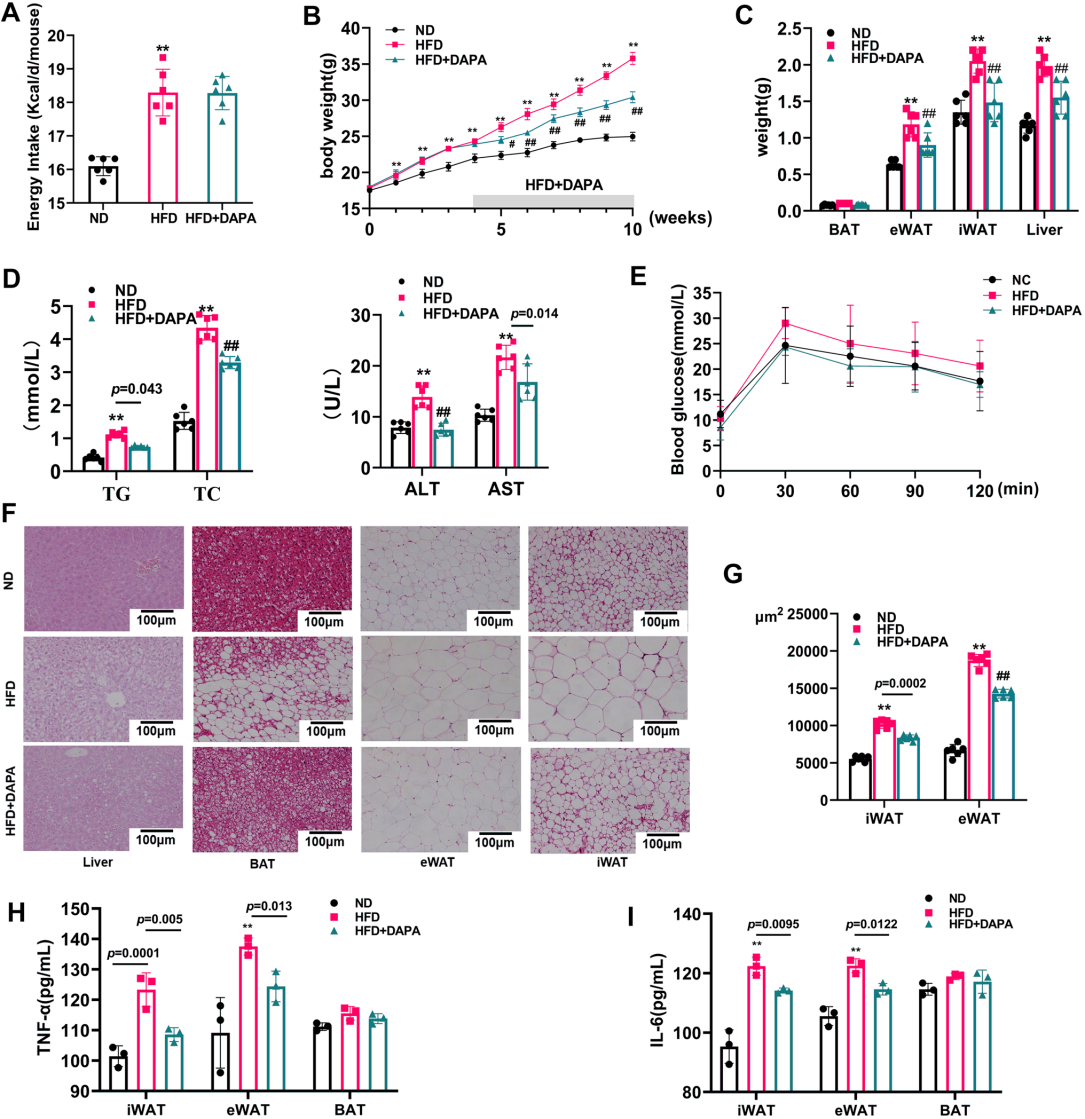
Effects of DAPA on HFD-induced obesity in C57BL/6 mice. After being fed a high-fat diet for 4 weeks, mice were injected with or without DAPA. Subsequently, **A** energy intake; **B** body weight was measured; **C** tissue weights of eWAT, iWAT, BAT, and liver were determined; **D** levels of ALT, AST, TC, and TG in plasma were measured for each group; **E** a glucose tolerance test was conducted; **F** H&E staining of eWAT, iWAT, BAT, and liver was performed, and the size of adipocytes in WAT was quantified (magnification: 200 × , scale bar: 100.24 μm); **G** adipocyte size in WAT was quantified; **H** TNF-α content in adipose tissue; **I** IL-6 content in adipose tissue, and The data are presented as mean ± SD; **P < 0.0001 *vs.* ND group; ^##^*P* < 0.0001 *vs.* HFD group

To investigate the potential benefits of DAPA use for obesity treatment, we replicated the obesity model with a high-fat diet and applied DAPA intervention in addition to obesity treatment, and it had no effect on energy intake (Fig. 1A). The results showed that, compared with the ND group, the HFD group immediately exhibited a significant increase in body weight (Fig. 1B). Additionally, disruption of lipid metabolism and impaired liver function (Fig. 1D) were observed in the HFD group. These metabolic changes were evident in the increased weights of adipose tissues and the liver as well as in hepatocellular ballooning (Fig. 1C, F, G), and were very similar to those that occur at the onset of human metabolic diseases. Furthermore, we found that DAPA intervention significantly improved the aforementioned changes but had no significant effect on glucose tolerance (Fig. 1E). Finally, we evaluated the effect of DAPA on inflammation in three types of adipose tissue, and the results showed that DAPA intervention significantly reduced the levels of inflammatory factors in iWAT and eWAT of the HFD group, demonstrating anti-inflammatory properties (Fig. 1H, I). These results demonstrate the potential of DAPA to treat metabolic disorders caused by obesity.

### DAPA regulated the browning of WAT and the activation of BAT in vivo

**Fig. 2:**
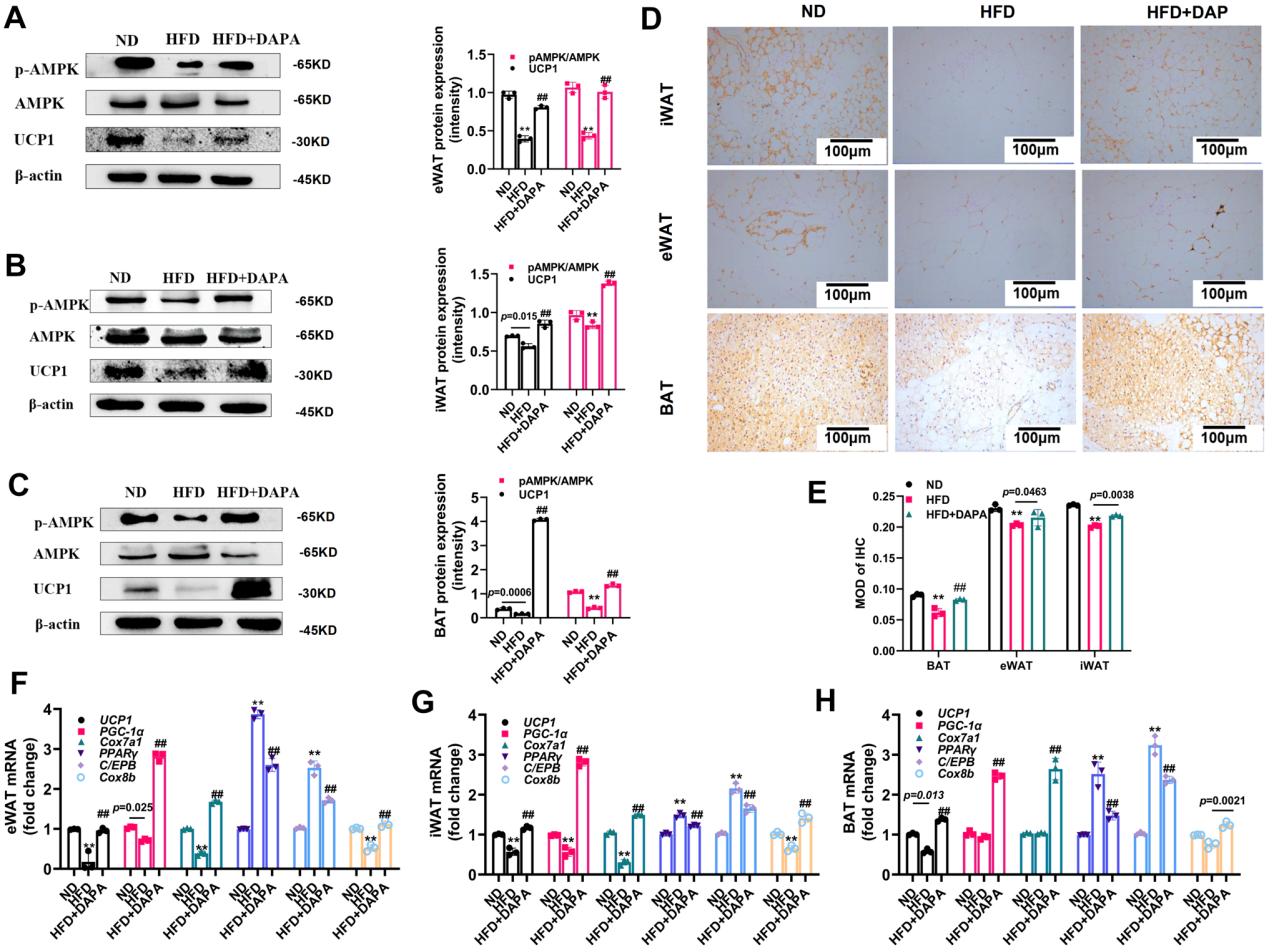
Effects of DAPA on “browning” of HFD-induced C57BL/6 mouse adipose tissue. After being fed a high-fat diet for 4 weeks, mice were injected with or without DAPA, and adipose tissue was collected after euthanasia to measure: **A** eWAT, **B** iWAT, and **C** BAT expression of thermogenic proteins; and **D** UCP1 immunoreactivity signals in eWAT, iWAT, and BAT tissues (brown color, magnification: 200 × , scale bar: 100 μm). **E** quantification of IHC. Additionally, qPCR was used to measure **F** thermogenic and lipogenic gene mRNA expression in eWAT, **G** iWAT, and **H** BAT. β-actin was used for normalization. The data are presented as mean ± SD; **P < 0.0001 *vs.* ND group; ^##^*P* < 0.0001 *vs.* HFD group

In the in vivo experiments, we observed a significant upregulation of UCP1 and p-AMPK expression in the iWAT, eWAT, and BAT of the obese mice following DAPA administration (Fig. 2A, B, C). Additionally, our assessment of UCP1 expression in adipose tissue revealed higher levels in the HFD + DAPA group (Fig. 2D, E). Subsequently, the qPCR was used to detect the effects of DAPA on adipogenic genes, including CCAAT/enhancer binding protein-α (*C/EBP-α*) and peroxisome proliferator-activated receptor γ (*PPARγ*), as well as thermogenesis-related genes such as *UCP1*, peroxisome proliferator-activated receptor γ coactivator-1α (*Pgc-1α*), Cytochrome C oxidase protein 7a1 (*Cox7a1*), and Cytochrome C oxidase subunit 8b (*Cox8b*). Figures 2F, G, and H show that DAPA downregulated the expression of adipogenic genes and upregulated the expression of thermogenesis-related genes. These findings suggest that DAPA enhanced WAT browning and BAT activation in vivo.

### DAPA promoted the browning process of 3T3-L1 cells

**Fig. 3:**
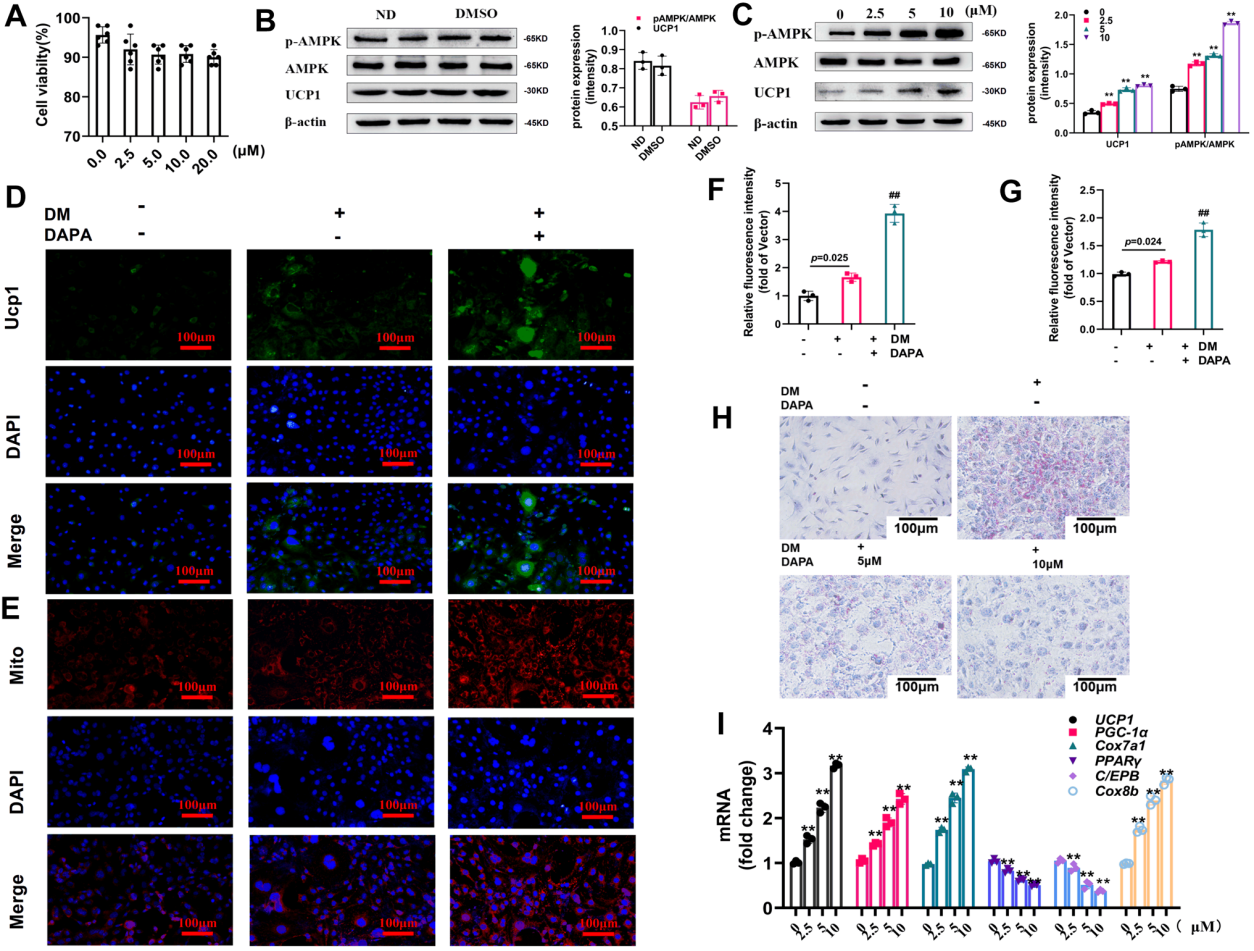
Effects of DAPA on differentiation of 3T3-L1 cells. Differentiated 3T3-L1 cells were treated with or without DAPA. Subsequently, **A** CCK-8 assay was performed; **B** expression of thermogenic protein in cells treated with DMSO were measured; **C** expression of thermogenic proteins was detected; **D** UCP1 antibody immunostaining was conducted, and DAPI counterstaining was used to observe cell nuclei (magnification: 200 × , scale bar: 100 μm); **E** mitochondrial staining with MitoTracker Red was performed (magnification: 200 × , scale bar: 100 μm); **F** Relative fluorescence intensity of UCP1 staining; **G** Relative fluorescence intensity of Mitochondrial staining; **H** neutral lipid staining with oil red O was conducted (magnification: 200 × , scale bar: 100 μm); and **I** mRNA expression of thermogenic and lipogenic genes were analyzed and normalized to β-actin. The data are presented as mean ± SD; **P < 0.0001 *vs.* control group, ^##^*P* < 0.0001 *vs.* DAPA treatment group. DM, differentiation medium

For the in vitro experiments, we treated 3T3-L1 cells with different concentrations of DAPA. The results presented in Fig. 3A show that different concentrations of DAPA (< 20 μM) had no discernible effects on cell viability. Furthermore, we excluded any potential influence of the vehicle, DMSO, on protein expression (Fig. 3B). Subsequently, we treated 3T3-L1 cells with different concentrations of DAPA and observed a dose-dependent increase in UCP1 and p-AMPK expression (Fig. 3C). Unless stated otherwise, all subsequent experiments employed a concentration of 10 μM DAPA. To further validate the effects of DAPA, immunofluorescence assays were performed, which revealed that DAPA increased UCP1 protein expression compared to that in the undifferentiated and differentiated groups (Fig. 3D, F). Because it is widely recognized that mature brown adipocytes are characterized by a higher mitochondrial content [23], we used MitoTracker probes to detect whether DAPA affected mitochondrial quantity in 3T3-L1 cells. The results indicated that the cellular mitochondrial content was elevated following DAPA treatment, as shown in Fig. 3E, G. Similarly, to verify the effects of DAPA on lipid accumulation through in vitro experiments, Oil Red O staining was performed. Figure 3H shows a significant reduction in lipid accumulation in the DAPA-treated cells. The qPCR results were consistent with the findings from the in vivo experiments, demonstrating that DAPA reduced the expression of adipogenesis-related genes and increased the expression of thermogenesis-related genes (Fig. 3I).

### DAPA regulated “browning” by promoting the FGFR1/KLB complex

**Fig. 4:**
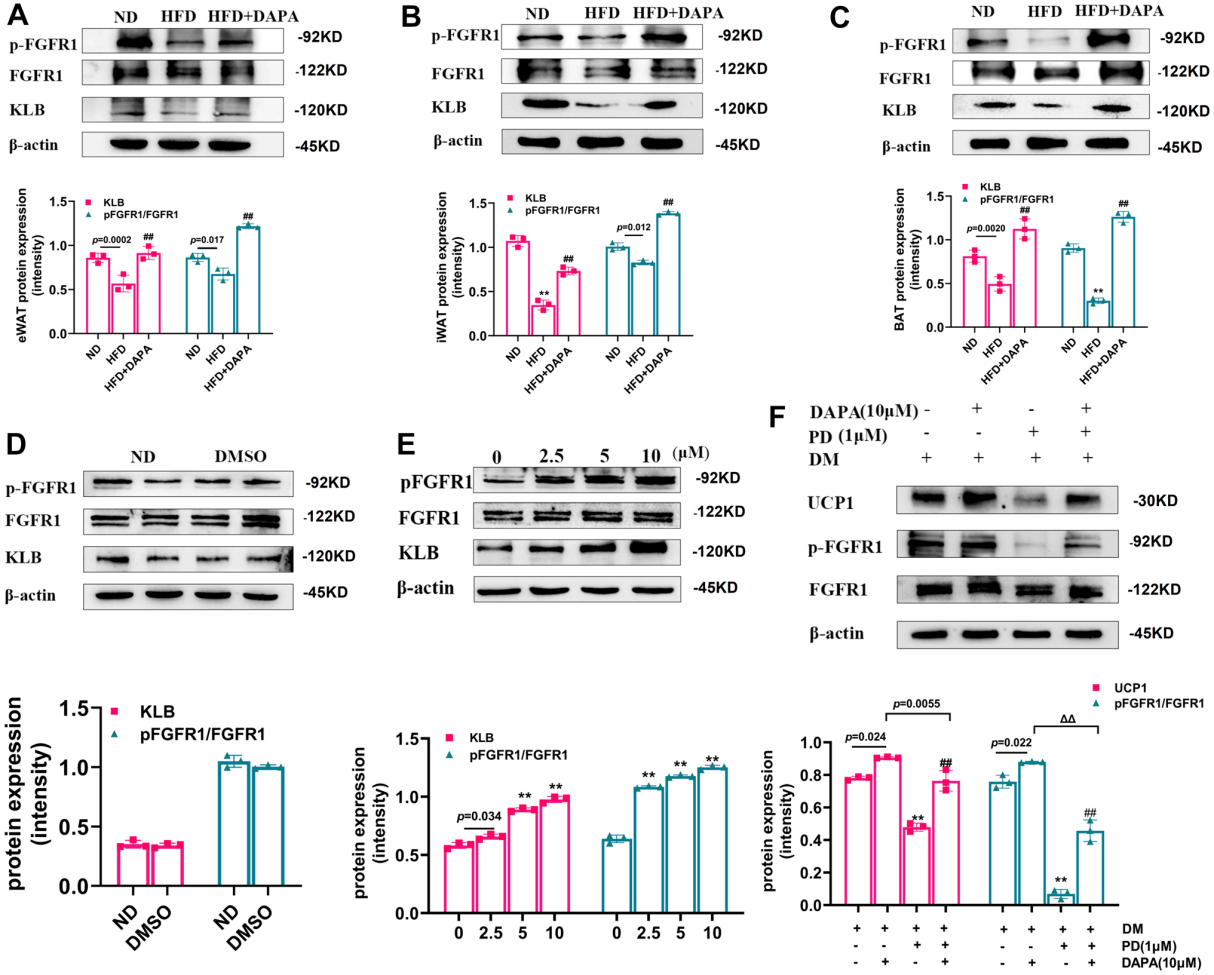
Effects of DAPA on FGFR1/KLB complex in vitro and in vivo. After feeding mice a high-fat diet for 4 weeks, DAPA was either injected or not injected, and the mice were sacrificed. p-FGFR1 and KLB protein expressions were detected by western blot in eWAT (**A**), iWAT (**B**), and BAT (**C**) of each group of mice; 3T3-L1 cells induced to differentiate were treated or not treated with DAPA. p-FGFR1 and KLB protein expression were measured after treatment with DMSO (**D**); protein expression of p-FGFR1 and KLB after DAPA treatment (**E**); After treatment with FGFR1 receptor inhibitor PD, the expression of pFGFR1, and heat shock protein were measured (**F**). Data are presented as mean ± SD; **P < 0.0001 *vs.* ND group; ^##^*P* < 0.0001 *vs.* HFD group; **P < 0.0001 vs. control group; ^##^*P* < 0.0001 vs. PD treatment group; ^ΔΔ^*P* < 0.0001 vs. DAPA treatment group. DM: differentiation medium.

The action of FGF21 in target organs requires binding with FGFR and the co-factor KLB, and fibroblast growth factor receptors 1 (FGFR1) played a major role in energy expenditure [24]. Therefore, to explore the effects of DAPA, we performed western blot analysis and observed that HFD suppressed the expression of, p-FGFR1, and KLB proteins; however, DAPA treatment restored their protein levels (Fig. 4A, B, C). These findings imply that DAPA enhances FGF21’s effectiveness in combating obesity-induced resistance by increasing the expression of the complex protein formed by p-FGFR1 and KLB. Consistent with the in vivo experiments, we first showed that DMSO did not affect the expression of p-FGFR1 and KLB(Fig. 4D). Subsequently, we evaluated the effects of DAPA treatment on key molecules involved in FGF21 signaling in 3T3-L1 cells. Our results indicated that DAPA increased the protein expression of p-FGFR1, and its co-receptor KLB in a dose-dependent manner (Fig. 4E). To further explore the involvement of FGFR1 in the “browning” process of 3T3-L1 cells, we used the FGFR1 receptor inhibitor, PD. Western blot analysis showed that, after inhibitor treatment, the protein expression of p-FGFR1 decreased, while the protein concentration of UCP1 was also reduced simultaneously (Fig. 4F), indicating the important role of FGFR1 in this process. In conclusion, our study provided evidence that DAPA treatment facilitates the “browning” of adipose tissue via activation of the FGFR1 signaling pathway.

### DAPA regulated “browning” through the FGFR1-LKB1-AMPK pathway

**Fig. 5:**
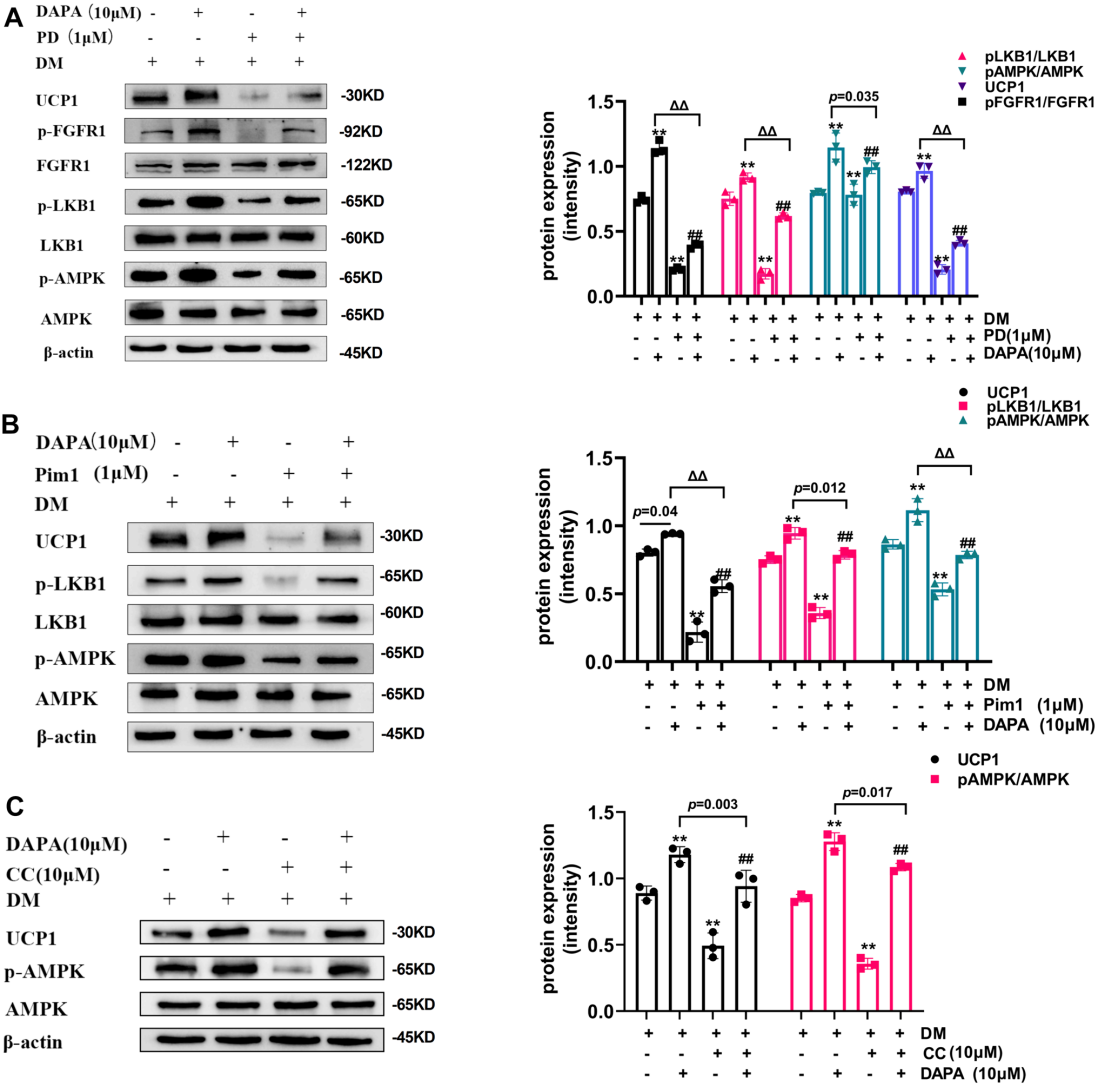
Effects of DAPA on AMPK pathway in 3T3-L1 cells. After treatment with PD, Pim1, CC, and treatment with DAPA, heat shock protein levels and the protein expression of p-FGFR1, p-LKB1, and p-AMPK were measured (**A**). Heat shock protein expression and the protein expression of p-LKB1, and p-AMPK were also measured (**B**). Heat shock protein expression and the protein expression of p-AMPK were also measured (**C**). Data are presented as mean ± SD; ^**^P < 0.0001 vs. control group; ^##^*P* < 0.0001 vs. inhibitor group; ^ΔΔ^*P* < 0.0001 vs. DAPA treatment group, DM: differentiation medium

In previous experiments, we established that DAPA increased the content of KLB on one hand, and on the other hand, it promoted the activation of p-FGFR1, enabling promote “browning”. To elucidate the underlying mechanisms involved in this process, we conducted further investigations using specific inhibitors in 3T3-L1 cells. Specifically, we used the FGFR1 receptor inhibitor, PD, to inhibit FGFR1 activity in 3T3-L1 cells. As shown in Fig. 5A, PD treatment significantly inhibited the protein expression of p-FGFR1, p-LKB1, p-AMPK, and UCP1. We also used the LKB1 inhibitor, Pim1, to interfere with LKB1 function in 3T3-L1 cells (Fig. 5B). Notably, the protein expression levels of p-LKB1, p-AMPK, and UCP1 were all significantly reduced by Pim1 treatment. Finally, we used the AMPK inhibitor, CC, which led to a decrease in the protein expression of p-AMPK and UCP1 (Fig. 5C). Importantly, DAPA reversed the inhibitory effects of both FGFR1, LKB1, and AMPK inhibitors. Collectively, our findings provided compelling evidence that the FGFR1-LKB1-AMPK pathway may be one of the mechanisms through which DAPA regulates the “browning” of adipose tissue.

## Discussion

The activation of BAT or induction of WAT “browning” has been shown to improve obesity and obesity-related metabolic syndrome [25]. Consequently, the identification of safe and effective approaches to promote browning has become an important focus of subsequent studies. In this study, we preliminarily validated the protective effect of DAPA on obesity and related metabolic disorders. DAPA, as the first approved SGLT2 inhibitor on the market, has a lower risk of inducing hypoglycemia compared to other traditional antidiabetic drugs [26]. Despite the lack of relevant data on this class of drugs, they can only be approved as second or third-line treatment options. Our findings provided some theoretical basis for the clinical application of SGLT2 inhibitors. BAT is considered to have sexual dimorphism [27], as studies in humans and animals have shown that female mice and women exhibit stronger energy expenditure capabilities in BAT, which is closely related to estrogen [28, 29]. Considering the gender differences in elucidating the regulatory effect of DAPA in promoting the “browning” of adipose tissue on metabolic disorders is also meaningful. Additionally, the application of various obesity models, such as ob/ob mice [30], is equally important in clarifying the role of DAPA in adipose tissue. However, it is undeniable that our study using a high-fat diet-induced obese male mouse model excluded the influence of gender and genetic factors on adipose tissue, making it more convincing to preliminarily clarify the role of DAPA in the “browning” of adipose tissue.

It is well known that SGLT2 inhibitors primarily lower blood sugar by inhibiting renal glucose reabsorption [31]. The coding genes for SGLT1 and SGLT2 are Scl5a1 and Scl5a2, respectively. Research indicates that in T2DM patients using SGLT2 inhibitors, the expression of the Scl5a1 gene in the pancreatic islets remains elevated while Scl5a2 gene expression is lower. This is in line with increased glucagon gene expression, suggesting that SGLT1/2 may play different roles in obesity, warranting further exploration [32]. Although there are no reports explicitly stating the direct relationship between SGLT2 and adipose tissue, As pointed out by Xuping Yang et al. [33], Canagliflozin can directly affect primary adipocytes, promoting mitochondrial biogenesis and thermogenesis. Our findings also confirmed that DAPA could activate BAT, promote “browning” of WAT, and increase fat tissue thermogenesis. While DAPA acting on SGLT2 may be involved in the body’s glucose and lipid metabolism processes, it cannot be denied that DAPA’s improvement in obesity and metabolic disorders partly stems from the “browning” of WAT, consistent with reports on other SGLT2 inhibitors [34, 35]. Due to the lack of the Oxymax Lab Animal Monitoring System, we were unable to record energy expenditure, which is a limitation on our part. Simon A. et al. point out that SGLT2 inhibitors do not activate AMPK in adipose tissue during acute treatment, which contradicts our experimental results. We speculate that this may be related to the duration of treatment. Our intervention is a short-term treatment, but for a medication, the body’s tolerance to long-term use, side effects, and maintenance of effectiveness are all key concerns. Long-term studies will help us clarify the effects of DAPA on the body, whether positive or negative, and this is worth exploring. Our study encountered a surprising result where DAPA did not significantly improve glucose tolerance in mice, possibly due to limitations in the HFD model’s glucose increase aspect [36]. However, this does not negate DAPA’s role in improving insulin resistance in the body [32]. Additionally, our research found that DAPA can improve WAT inflammation, suggesting a potential link between DAPA’s improvement of metabolic disorders and inflammation, which warrants further exploration.

FGF21 is a key factor in the fibroblast growth factor family involved in glucose and lipid metabolism, controlling blood sugar, lipid levels, and weight reduction [37]. Liang Xu et al. [34] find that Empagliflozin can increase the levels of FGF21 in the liver and systemic circulation, and FGF21 can promote the “browning” of WAT and directly act on BAT. This suggests that SGLT2 inhibitors promoting the utilization of FGF21 by adipose tissue is one possible mechanism for inducing “browning,” although the specific mechanism is not yet fully understood. Yingzhe Xiong et al. [38] observe that elevated FGF21 in obese individuals does not exhibit its protective effects, possibly due to reduced FGFR1 and KLB receptors, leading to a loss of FGF21 signaling cascade. Our research indicated that DAPA could activate the FGF21 signaling cascade, promote adipose tissue “browning,” and suggest that SGLT2 inhibitors may act as FGFR1 agonists, demonstrating for the first time the association between SGLT2 inhibitors and FGFR1. Given the superior role of FGF21 in improving metabolic disorders, developing FGF21 analogs is also a strategy to combat obesity. Existing long-acting FGF21 analogs essentially act as FGFR agonists but have not yet been approved for clinical use [39]. Our findings enriched the clinical applications of SGLT inhibitors. AMPK, as an energy regulator, is closely related to the FGF21 signaling cascade and plays a crucial role in regulating energy in the body [40]. Previous reports have shown that Momordica charantia enhances liver FGF21 and AMPK/Sirt1 signaling to alleviate hepatic steatosis in mice [41]. Our study suggests that the potential mechanism by which SGLT2 inhibitors promote the “browning” of WAT involves the FGFR1-LKB1-AMPK signaling pathway, but correlation does not imply causation. In subsequent developments, we will generate FGFR1 gene knockout mice to elucidate the role of FGFR1 signaling cascade in SGLT inhibitors promoting WAT “browning” and the molecular mechanisms involved.

In summary, our findings suggest that DAPA effectively improved metabolic disorders caused by HFD. The beneficial effects of DAPA were attributed to its ability to induce “browning” of adipose tissue, and the FGFR1-LKB1-AMPK signaling pathway might play an important role in mediating this browning process.

## Data Availability

The data that support the findings of this study are available from the authors but restrictions apply to the availability of these data, which were used under license from the Natural History Museum (London) for the current study, and so are not publicly available. Data are, however, available from the authors upon reasonable request and with permission from the Centre for Human Evolution Studies at the Natural History Museum. Example from: huiwenwu@sxmu.edu.cn.

## References

[1] Unamuno X, Gomez-Ambrosi J, Rodriguez A, Becerril S, Fruhbeck G, Catalan V (2018) Adipokine dysregulation and adipose tissue inflammation in human obesity. Eur J Clin Invest 48(9):e12997. 10.1111/eci.12997 29995306 10.1111/eci.12997

[2] Frigolet ME, Gutierrez-Aguilar R (2020) The colors of adipose tissue. Gac Med Mex 156(2):142–149. 10.24875/GMM.M20000356 32285854 10.24875/GMM.M20000356

[3] Fernandez-Verdejo R, Marlatt KL, Ravussin E, Galgani JE (2019) Contribution of brown adipose tissue to human energy metabolism. Mol Aspects Med 68:82–89. 10.1016/j.mam.2019.07.003 31306668 10.1016/j.mam.2019.07.003 PMC7112661

[4] Jiang H, Ding X, Cao Y, Wang H, Zeng W (2017) Dense intra-adipose sympathetic arborizations are essential for cold-induced beiging of mouse white adipose tissue. Cell Metab 26(4):686–692. 10.1016/j.cmet.2017.08.016 28918935 10.1016/j.cmet.2017.08.016

[5] van der Vaart JI, Boon MR, Houtkooper RH (2021) The role of AMPK signaling in brown adipose tissue activation. Cells 10(5):1122. 10.3390/cells10051122 34066631 10.3390/cells10051122 PMC8148517

[6] Suzuki M, Uehara Y, Motomura-Matsuzaka K, Oki J, Koyama Y, Kimura M, Asada M, Komi-Kuramochi A, Oka S, Imamura T (2008) betaKlotho is required for fibroblast growth factor (FGF) 21 signaling through FGF receptor (FGFR) 1c and FGFR3c. Mol Endocrinol 22(4):1006–1014. 10.1210/me.2007-0313 18187602 10.1210/me.2007-0313 PMC5419549

[7] Geng L, Liao B, Jin L, Huang Z, Triggle CR, Ding H, Zhang J, Huang Y, Lin Z, Xu A (2019) Exercise alleviates obesity-induced metabolic dysfunction via enhancing FGF21 sensitivity in adipose tissues. Cell Rep 26(10):2738–2752. 10.1016/j.celrep.2019.02.014 30840894 10.1016/j.celrep.2019.02.014

[8] Adams AC, Yang C, Coskun T, Cheng CC, Gimeno RE, Luo Y, Kharitonenkov A (2012) The breadth of FGF21’s metabolic actions are governed by FGFR1 in adipose tissue. Mol Metab 2(1):31–37. 10.1016/j.molmet.2012.08.007 24024127 10.1016/j.molmet.2012.08.007 PMC3757657

[9] Yang W, Liu L, Wei Y, Fang C, Zhou F, Chen J, Han Q, Huang M, Tan X, Liu Q, Pan Q, Zhang L, Lei X, Li L (2019) Exercise ameliorates the FGF21-adiponectin axis impairment in diet-induced obese mice. Endocr Connect 8(5):596–604. 10.1530/EC-19-0034 30978696 10.1530/EC-19-0034 PMC6510890

[10] Lelliott CJ, Ahnmark A, Admyre T, Ahlstedt I, Irving L, Keyes F, Patterson L, Mumphrey MB, Bjursell M, Gorman T, Bohlooly YM, Buchanan A, Harrison P, Vaughan T, Berthoud HR, Linden D (2014) Monoclonal antibody targeting of fibroblast growth factor receptor 1c ameliorates obesity and glucose intolerance via central mechanisms. PLoS ONE 9(11):e112109. 10.1371/journal.pone.0112109 25427253 10.1371/journal.pone.0112109 PMC4245083

[11] Nedergaard J, Cannon B (2014) The browning of white adipose tissue: some burning issues. Cell Metab 20(3):396–407. 10.1016/j.cmet.2014.07.005 25127354 10.1016/j.cmet.2014.07.005

[12] Swe MT, Thongnak L, Jaikumkao K, Pongchaidecha A, Chatsudthipong V, Lungkaphin A (2019) Dapagliflozin not only improves hepatic injury and pancreatic endoplasmic reticulum stress, but also induces hepatic gluconeogenic enzymes expression in obese rats. Clin Sci (Lond) 133(23):2415–2430. 10.1042/CS20190863 31769484 10.1042/CS20190863

[13] Zugner E, Yang HC, Kotzbeck P, Boulgaropoulos B, Sourij H, Hagvall S, Elmore CS, Esterline R, Moosmang S, Oscarsson J, Pieber TR, Peng XR, Magnes C (2022) Differential in vitro effects of SGLT2 inhibitors on mitochondrial oxidative phosphorylation, glucose uptake and cell metabolism. Int J Mol Sci 23(14):7966. 10.3390/ijms23147966 35887308 10.3390/ijms23147966 PMC9319636

[14] Sato D, Nakamura T, Amarume J, Yano M, Umehara Y, Nishina A, Tsutsumi K, Feng Z, Kusunoki M (2022) Effects of dapagliflozin on adipose and liver fatty acid composition and mRNA expression involved in lipid metabolism in high-fat-fed rats. Endocr Metab Immune Disord Drug Targets 22(9):944–953. 10.2174/1871530322666220307153618 35255800 10.2174/1871530322666220307153618

[15] Han T, Fan Y, Gao J, Fatima M, Zhang Y, Ding Y, Bai L, Wang C (2021) Sodium glucose cotransporter 2 inhibitor dapagliflozin depressed adiposity and ameliorated hepatic steatosis in high-fat diet induced obese mice. Adipocyte 10(1):446–455. 10.1080/21623945.2021.1979277 34550043 10.1080/21623945.2021.1979277 PMC8475578

[16] Sa-Nguanmoo P, Tanajak P, Kerdphoo S, Jaiwongkam T, Pratchayasakul W, Chattipakorn N, Chattipakorn SC (2017) SGLT2-inhibitor and DPP-4 inhibitor improve brain function via attenuating mitochondrial dysfunction, insulin resistance, inflammation, and apoptosis in HFD-induced obese rats. Toxicol Appl Pharmacol 333:43–50. 10.1016/j.taap.2017.08.005 28807765 10.1016/j.taap.2017.08.005

[17] Steinberg GR, Carling D (2019) AMP-activated protein kinase: the current landscape for drug development. Nat Rev Drug Discov 18(7):527–551. 10.1038/s41573-019-0019-2 30867601 10.1038/s41573-019-0019-2

[18] Lopez M, Varela L, Vazquez MJ, Rodriguez-Cuenca S, Gonzalez CR, Velagapudi VR, Morgan DA, Schoenmakers E, Agassandian K, Lage R, Martinez de Morentin PB, Tovar S, Nogueiras R, Carling D, Lelliott C, Gallego R, Oresic M, Chatterjee K, Saha AK, Rahmouni K, Dieguez C, Vidal-Puig A (2010) Hypothalamic AMPK and fatty acid metabolism mediate thyroid regulation of energy balance. Nat Med 16(9):1001–1008. 10.1038/nm.2207 20802499 10.1038/nm.2207 PMC2935934

[19] Shaw RJ (2009) LKB1 and AMP-activated protein kinase control of mTOR signalling and growth. Acta Physiol (Oxf) 196(1):65–80. 10.1111/j.1748-1716.2009.01972.x 19245654 10.1111/j.1748-1716.2009.01972.x PMC2760308

[20] Luo J, Sun P, Wang Y, Chen Y, Niu Y, Ding Y, Xu N, Zhang Y, Xie W (2021) Dapagliflozin attenuates steatosis in livers of high-fat diet-induced mice and oleic acid-treated L02 cells via regulating AMPK/mTOR pathway. Eur J Pharmacol 907:174304. 10.1016/j.ejphar.2021.174304 34224699 10.1016/j.ejphar.2021.174304

[21] Swe MT, Thongnak L, Jaikumkao K, Pongchaidecha A, Chatsudthipong V, Lungkaphin A (2020) Dapagliflozin attenuates renal gluconeogenic enzyme expression in obese rats. J Endocrinol 245(2):193–205. 10.1530/JOE-19-0480 32092034 10.1530/JOE-19-0480

[22] Yoshioka H, Ohishi R, Hirose Y, Torii-Goto A, Park SJ, Miura N, Yoshikawa M (2019) Chronopharmacology of dapagliflozin-induced antihyperglycemic effects in C57BL/6J mice. Obes Res Clin Pract 13(5):505–510. 10.1016/j.orcp.2019.08.001 31466832 10.1016/j.orcp.2019.08.001

[23] Gallardo-Montejano VI, Yang C, Hahner L, McAfee JL, Johnson JA, Holland WL, Fernandez-Valdivia R, Bickel PE (2021) Perilipin 5 links mitochondrial uncoupled respiration in brown fat to healthy white fat remodeling and systemic glucose tolerance. Nat Commun 12(1):3320. 10.1038/s41467-021-23601-2 34083525 10.1038/s41467-021-23601-2 PMC8175597

[24] Kolumam G, Chen MZ, Tong R, Zavala-Solorio J, Kates L, van Bruggen N, Ross J, Wyatt SK, Gandham VD, Carano RA, Dunshee DR, Wu AL, Haley B, Anderson K, Warming S, Rairdan XY, Lewin-Koh N, Zhang Y, Gutierrez J, Baruch A, Gelzleichter TR, Stevens D, Rajan S, Bainbridge TW, Vernes JM, Meng YG, Ziai J, Soriano RH, Brauer MJ, Chen Y, Stawicki S, Kim HS, Comps-Agrar L, Luis E, Spiess C, Wu Y, Ernst JA, McGuinness OP, Peterson AS, Sonoda J (2015) Sustained brown fat stimulation and insulin sensitization by a humanized bispecific antibody agonist for fibroblast growth factor receptor 1/betaKlotho complex. EBioMedicine 2(7):730–743. 10.1016/j.ebiom.2015.05.028 26288846 10.1016/j.ebiom.2015.05.028 PMC4534681

[25] Jelcic J, Korsic M (2009) Obesity as a medical and public health problem. Lijec Vjesn 131(9–10):279–285 20030293

[26] Dhillon S (2019) Dapagliflozin: a review in type 2 diabetes. Drugs 79(10):1135–1146. 10.1007/s40265-019-01148-3 31236801 10.1007/s40265-019-01148-3 PMC6879440

[27] Kaikaew K, Grefhorst A, Visser JA (2021) Sex differences in brown adipose tissue function: sex hormones, glucocorticoids, and their crosstalk. Front Endocrinol (Lausanne) 12:652444. 10.3389/fendo.2021.652444 33927694 10.3389/fendo.2021.652444 PMC8078866

[28] Rodriguez AM, Quevedo-Coli S, Roca P, Palou A (2001) Sex-dependent dietary obesity, induction of UCPs, and leptin expression in rat adipose tissues. Obes Res 9(9):579–588. 10.1038/oby.2001.75 11557839 10.1038/oby.2001.75

[29] Brendle C, Werner MK, Schmadl M, la Fougere C, Nikolaou K, Stefan N, Pfannenberg C (2018) Correlation of brown adipose tissue with other body fat compartments and patient characteristics: a retrospective analysis in a large patient cohort using PET/CT. Acad Radiol 25(1):102–110. 10.1016/j.acra.2017.09.007 29108812 10.1016/j.acra.2017.09.007

[30] Suriano F, Vieira-Silva S, Falony G, Roumain M, Paquot A, Pelicaen R, Regnier M, Delzenne NM, Raes J, Muccioli GG, Van Hul M, Cani PD (2021) Novel insights into the genetically obese (ob/ob) and diabetic (db/db) mice: two sides of the same coin. Microbiome 9(1):147. 10.1186/s40168-021-01097-8 34183063 10.1186/s40168-021-01097-8 PMC8240277

[31] Sodium-Glucose Cotransporter-2 (SGLT2) Inhibitors. *LiverTox: Clinical and Research Information on Drug-Induced Liver Injury*. Bethesda (MD); 2012. 31643176

[32] Bonner C, Kerr-Conte J, Gmyr V, Queniat G, Moerman E, Thevenet J, Beaucamps C, Delalleau N, Popescu I, Malaisse WJ, Sener A, Deprez B, Abderrahmani A, Staels B, Pattou F (2015) Inhibition of the glucose transporter SGLT2 with dapagliflozin in pancreatic alpha cells triggers glucagon secretion. Nat Med 21(5):512–517. 10.1038/nm.3828 25894829 10.1038/nm.3828

[33] Yang X, Liu Q, Li Y, Tang Q, Wu T, Chen L, Pu S, Zhao Y, Zhang G, Huang C, Zhang J, Zhang Z, Huang Y, Zou M, Shi X, Jiang W, Wang R, He J (2020) The diabetes medication canagliflozin promotes mitochondrial remodelling of adipocyte via the AMPK-Sirt1-Pgc-1alpha signalling pathway. Adipocyte 9(1):484–494. 10.1080/21623945.2020.1807850 32835596 10.1080/21623945.2020.1807850 PMC7469612

[34] Xu L, Nagata N, Nagashimada M, Zhuge F, Ni Y, Chen G, Mayoux E, Kaneko S, Ota T (2017) SGLT2 inhibition by empagliflozin promotes fat utilization and browning and attenuates inflammation and insulin resistance by polarizing M2 macrophages in diet-induced obese mice. EBioMedicine 20:137–149. 10.1016/j.ebiom.2017.05.028 28579299 10.1016/j.ebiom.2017.05.028 PMC5478253

[35] Xu L, Nagata N, Chen G, Nagashimada M, Zhuge F, Ni Y, Sakai Y, Kaneko S, Ota T (2019) Empagliflozin reverses obesity and insulin resistance through fat browning and alternative macrophage activation in mice fed a high-fat diet. BMJ Open Diabetes Res Care 7(1):e000783. 10.1136/bmjdrc-2019-000783 31749970 10.1136/bmjdrc-2019-000783 PMC6827766

[36] Haas B, Eckstein N, Pfeifer V, Mayer P, Hass MD (2014) Efficacy, safety and regulatory status of SGLT2 inhibitors: focus on canagliflozin. Nutr Diabetes 4(11):e143. 10.1038/nutd.2014.40 25365416 10.1038/nutd.2014.40 PMC4259905

[37] Geng L, Lam KSL, Xu A (2020) The therapeutic potential of FGF21 in metabolic diseases: from bench to clinic. Nat Rev Endocrinol 16(11):654–667. 10.1038/s41574-020-0386-0 32764725 10.1038/s41574-020-0386-0

[38] Xiong Y, Chen Y, Liu Y, Zhang B (2020) Moderate-intensity continuous training improves FGF21 and KLB expression in obese mice. Biochemistry (Mosc) 85(8):938–946. 10.1134/S000629792008009X 33045954 10.1134/S000629792008009X

[39] Yang R, Xu A, Kharitonenkov A (2022) Another kid on the block: long-acting FGF21 analogue to treat dyslipidemia and fatty liver. J Clin Endocrinol Metab 107(1):e417–e419. 10.1210/clinem/dgab686 34529079 10.1210/clinem/dgab686 PMC8684496

[40] Salminen A, Kauppinen A, Kaarniranta K (2017) FGF21 activates AMPK signaling: impact on metabolic regulation and the aging process. J Mol Med (Berl) 95(2):123–131. 10.1007/s00109-016-1477-1 27678528 10.1007/s00109-016-1477-1

[41] Yu Y, Zhang XH, Ebersole B, Ribnicky D, Wang ZQ (2013) Bitter melon extract attenuating hepatic steatosis may be mediated by FGF21 and AMPK/Sirt1 signaling in mice. Sci Rep 3:3142. 10.1038/srep03142 24189525 10.1038/srep03142 PMC3912441

